# Acknowledgement to the Reviewers of GMS Journal for Medical Education

**DOI:** 10.3205/zma001148

**Published:** 2018-02-15

**Authors:** Martin R. Fischer, Götz Fabry

**Affiliations:** 1 GMS Journal for Medical Education, Chief Editor, Erlangen, Germany; 2 Klinikum der Universität München, Institut für Didaktik und Ausbildungsforschung in der Medizin, München, Germany; 3 GMS Journal for Medical Education, Assistant Chief Editor, Erlangen, Germany; 4 Albert-Ludwig-Universität Freiburg, Medizinische Fakultät, Abteilung für Medizinische Psychologie und Soziologie, Freiburg, Germany

## Acknowledgement

We gratefully acknowledge the valuable contribution of the following peer reviewers, who supported our Journal in 2017. We are looking forward to continuing our collaboration!

## Reviewers in alphabetical order

- Helmut Ahrens, Münster
- Obada al Halabi, Heidelberg
- Simone Alvarez, Heidelberg
- Stephanie Alvino, Bonn
- Matthias Angstwurm, München
- Timo Astfalk, Rostock
- Cadja Bachmann, Bern
- Anne Barzel, Hamburg
- Franziska Bässler, Heidelberg
- Daniel Bauer, Bern
- Pascal Berberat, München
- Antje Bergmann, Dresden
- Silke Biller, Basel
- Stephan Birk, Berlin
- Monika Bobbert, Münster
- Martin Boeker, Freiburg
- Klaus Böhme, Freiburg
- Patrick Boldt, Düsseldorf
- Hans Martin Bosse, Düsseldorf
- Myrto Boukovala, München
- Barbara Braun, Heidelberg
- Jan Breckwoldt, Zürich
- Irene Brunk, Berlin
- Till Bugaj, Heidelberg
- Andreas Burger, Bochum
- Beatrice Buss, Bern
- Helena de Carvalho Gomes, Stockholm
- Antje Degel, Berlin
- Peter Dieter, Dresden
- Diana Dolmans, Maastricht
- Markus Dürsch, Göttingen
- Julia Eckel, Mannheim
- Jan P. Ehlers, Witten
- Nurith Epstein, München
- Volkhard Fischer, Hannover
- Johannes Forster, Merzhausen
- Petra Ganschow, München
- Waltraud Georg, Zürich
- Marianne Giesler, Freiburg
- Katja Götz, Lübeck
- Matthäus Grasl, Wien
- Markus Gulich, Ulm
- Jennifer Guse, Hamburg
- Petra Hahn, Freiburg
- Eckhart G. Hahn, Erlangen
- Wolfgang Hampe, Hamburg
- Sigrid Harendza, Hamburg
- Cordula Harter, Heidelberg
- Wolf Hautz, Bern
- Inga Hege, München
- Axel Heller, Dresden
- Gunther Hempel, Leipzig
- Michael Hempel, Leipzig
- Jan Hense, Giessen
- Anke Hollinderbäumer, Mainz
- Anita Holzinger, Wien
- Thorsten Hornung, Bonn
- Marion Huber, Winterthur
- Markus Huber-Lang, Ulm
- Bert Huenges, Bochum
- Peter Iblher, Lübeck
- Jonas Johannink, Berlin
- Martina Kadmon, Augsburg
- Hanna Kaduszkiewicz, Kiel
- Yassin Karay, Köln
- Jan Kiesewetter, München
- Volker Köllner, Teltow
- Sarah König, Würzburg
- Eduard Kraft, München
- Markus Krautter, Stuttgart
- Felicitas Lahner, Bern
- Ronny Lehmann, Heidelberg
- Beate Lenk, Erfurt
- Martin Lischka, Wien
- Sabine Ludwig, München
- Jörg Marienhagen, Regensburg
- Maren März, Leipzig
- Jan Matthes, Köln
- Hannes Mayerl, Graz
- Martina Michaelis, Freiburg
- Andre Mihaljevic, Heidelberg
- Andreas Möltner, Heidelberg
- Brigitte Müller-Hilke, Rostock
- Elisabeth Narciß, Heidelberg
- Felix Nickel, Heidelberg
- Michael Noll-Hussong, Ulm
- Zineb Miriam Nouns, Berlin
- Frank Nürnberger, Frankfurt/Main
- Wolfgang Paik, München
- Ricardo Patricio Peréz Anderson, München
- Tim Peters, Bochum
- Harm Peters, Berlin
- Karen Pierer, Innsbruck
- Reinhard Putz, München
- Sabine Ramspott, München
- Oliver Rauprich, München
- Annett Reißhauer, Berlin
- Christopher Roberts, Sydney
- Katrin Rockenbauch, Leipzig
- Marco Roos, Erlangen
- Miriam Rüsseler, Frankfurt/Main
- Stefanie Sasaki-Sellmer, Magdeburg
- Thomas Sauter, Bern
- Thorsten Schäfer, Bochum
- Stefan Schauber, Oslo
- Jörg Schelling, München
- Theresa Scherer, Bern
- Ralf Schmidmaier, München
- Anita Schmidt, Erlangen
- Tabea Schmidt-Ott, London
- Petra Schnell-Inderst, Hall
- Gabriele Schroeder, Zürich
- Henrike Schulze, Hannover
- Katrin Schüttpelz-Brauns, Mannheim
- Hanna Seidling, Heidelberg
- Anne Simmenroth, Göttingen
- Melanie Simon, Aachen
- Andreas Sönnichsen, Witten
- Bernhard Steinweg, Bonn
- Sylvère Störmann, München
- Christoph Stosch, Köln
- Irmgard Streitlein-Böhme, Freiburg
- Nils Thiessen, Bonn
- Daniel Tolks, München
- Marie-Laurance Tremblay, Quebec
- Hendrik van den Bussche, Hamburg
- Michaela Wagner-Menghin, Wien
- Christiane Walter, Heidelberg
- Martin Weigl, München
- Mark Weinert, München
- Stefanie Wiemer, Leipzig
- Mario Wijnen-Meijer, Utrecht
- Ulrich Woermann, Bern
- Anne Wolowski, Münster
- Danmei Zhang, München
- Stine Ziegler, Hamburg
- Jan Zottmann, München
- Michaela Zupanic, Witten

